# Cognitive abilities and psychosocial functioning in bipolar disorder: findings from the BIPLONG study

**DOI:** 10.3389/fnhum.2025.1479648

**Published:** 2025-03-20

**Authors:** Elena M. D. Schönthaler, Nina Dalkner, Tatjana Stross, Susanne Bengesser, Julia Ilic, Frederike Fellendorf, Alexander Finner, Eva Fleischmann, Alfred Häussl, Johanna Georgi, Alexander Maget, Melanie Lenger, Annamaria Painold, Martina Platzer, Robert Queissner, Franziska Schmiedhofer, Stefan Smolle, Adelina Tmava-Berisha, Eva Z. Reininghaus

**Affiliations:** Clinical Division of Psychiatry and Psychotherapeutic Medicine, Medical University of Graz, Graz, Austria

**Keywords:** bipolar disorder, cognition, functioning, mood disorders, attention, memory, executive function

## Abstract

**Background:**

Bipolar disorder is associated with impairments in cognition and psychosocial functioning. Although these impairments occur frequently, often persist during euthymic times, and worsen quality of life, the impact of cognitive abilities on functioning has not yet been fully elucidated.

**Methods:**

The current study investigated the effects of cognitive domains (attention/psychomotor speed, verbal learning/memory, executive function) on psychosocial functioning cross-sectionally. Data from 210 euthymic individuals with bipolar disorder [101 female, 109 male; *M*_(age)_ = 44.47; *SD*_(age)_ = 14.25] were included into the analysis. A neurocognitive test battery was administered and the Global Assessment of Functioning was used to depict psychosocial functioning. Correlation analyses were conducted to observe the associations between functioning and the cognitive domains. Moreover, three hierarchical regression analyses were applied to predict functioning by each of the cognitive domains, while considering age, sex, and education as control variables.

**Results:**

Correlation analyses revealed that functioning was positively associated with attention/psychomotor speed and verbal learning/memory. However, the consecutive hierarchical regression analyses found that none of the cognitive domains were able predict functioning beyond the control variables age, sex, and education.

**Conclusion:**

Our findings indicate that greater abilities in the domains of attention/psychomotor speed and verbal learning/memory are associated with better functioning. However, this association can be explained by other relevant variables such as age or education, indicating that cognitive abilities are not the sole contributor of psychosocial functioning. Investigating other measurements of functioning or cognitive abilities could lead to different results. Nevertheless, promoting cognitive abilities and autonomy in daily life remains an important aspect of therapy in bipolar disorder.

## 1 Introduction

Bipolar disorder (BD) is a neuropsychiatric disorder characterized by recurring (hypo) manic and depressive episodes with an estimated prevalence of 1–2% worldwide (Blanco et al., 2017). Common symptoms of manic episodes include an expansive or irritated mood, highly elevated activity, decreased need for sleep, overconfidence, grandiosity, talkativeness, and impulse disinhibition (Carvalho et al., 2020). Contrary, depressive episodes encompass, among others, persistent sadness, loss of interest or pleasure, fatigue, sleep disturbances, and feelings of worthlessness or guilt (Dilling and Freyberger, 2019). In both mood states, cognitive deficits including attention, memory, and executive function impairments occur frequently and often persist during euthymia, especially in longer illness durations (Chen et al., 2023; Tsapekos et al., 2021). Due to these debilitating symptoms, BD was ranked as one of the 20 leading causes of disability worldwide (Anyayo et al., 2021; Arvilommi et al., 2022), and the worldwide prevalence of moderate to severe disability for BD is estimated at approximately 22 million individuals (Chen et al., 2019). One of the driving aspects of disability in BD is the low level of functioning, which differs slightly across mood states (Rosa et al., 2009; Van Der Voort et al., 2015) and serves as a key domain of illness outcome (Gitlin and Miklowitz, 2017), low quality of life, productivity loss (Kato et al., 2021), and suicidality (Rosa et al., 2008).

Functioning represents a multidimensional concept, including the ability to perform and engage in various life situations (World Health Organization, 2015), as well as managing aspects of the environment that help or hinder these life experiences (Chen et al., 2019). Over the past decades, the term global functioning, which subsumes the measurement of several types of functioning, has emerged (Aas, 2010). Global functioning encompasses, among others, psychosocial functioning (i.e., the ability to sustain social relationships and engage in activities of daily life; Michalak et al., 2010), occupational and educational functioning (i.e., the ability to perform work-related skills; Duarte et al., 2016), cognitive functioning (using cognitive function in real-world settings; Miskowiak et al., 2012), and physical functioning (e.g., mobility, dexterity, carrying out instrumental activities of daily living; World Health Organization, 2015). Individuals with BD often show low levels of functioning in these areas (Gitlin and Miklowitz, 2017; Sylvia et al., 2017), which affects them and their social structures (Baune and Malhi, 2015). For instance, they report severe work impairment (Boland et al., 2015; Marwaha et al., 2013), high unemployment rates (Holm et al., 2020), decreased social resources (Greenberg et al., 2014; Studart et al., 2015), and more barriers to higher education (Kruse and Oswal, 2018; Mojtabai et al., 2015). These functional impairments persist into symptomatic remission (Gitlin and Miklowitz, 2017; Léda-Rêgo et al., 2020), show great heterogeneity among individuals with BD (Solé et al., 2018), and lead to several difficulties in daily life (Chen et al., 2019). Since for many individuals with BD and their caregivers, functioning is at times more important than symptomatic outcome, much research has been dedicated to identifying its determinants. For instance, male sex, lower levels of education, lower socio-economic status, longer illness duration, a higher number of episodes, the presence of comorbid psychiatric disorders, and a greater current number of psychopharmacological medications have been found to predict functional impairment in BD (Burdick et al., 2022; Hower et al., 2019; Sanchez-Moreno et al., 2018; Wingo et al., 2010). Moreover, persistent subsyndromal symptoms, especially residual depressive symptoms, a history of psychosis (Bonnín et al., 2019; Bowie et al., 2010), and the number of admissions have been reported to have a negative impact on functioning (Sanchez-Moreno et al., 2017). Next to clinical and demographic variables, cognition has been found to be a predictor of functioning in BD in some studies (Burdick and Millett, 2021; Depp et al., 2012; Van Rheenen et al., 2019).

Cognition is usually conceptualized in terms of hierarchically ordered domains of cognitive function (i.e., the bottom domains refer to basic sensory and perceptual processes, and the top domains refer to executive function and cognitive control). The cognitive function domains encompass attention and concentration, memory, executive function, processing speed, and language/verbal skills (Harvey, 2019). In BD, a study revealed that 30–50% show impairments in cognition, predominantly in the areas of verbal memory, processing speed, executive function, attention, working memory, and social cognition (average illness duration: 15.61 years, average episode number: 11.28 episodes; Lima et al., 2019). Former studies reported that cognitive dysfunction is also related to demographic [e.g., age (Frías et al., 2017; Rabelo-da-Ponte et al., 2022; Mann-Wrobel et al., 2011), education (Rabelo-da-Ponte et al., 2022; Mann-Wrobel et al., 2011), sex (Martín-Parra et al., 2024)] and clinical outcomes [e.g., symptomatology; (Frías et al., 2017), number of hospitalizations (Rabelo-da-Ponte et al., 2022), illness duration (Mann-Wrobel et al., 2011), illness onset (Bortolato et al., 2015)]. Moreover, cognitively impaired individuals with BD were shown to display more functional disabilities than those without cognitive impairment (Jensen et al., 2016). The strength of the association between cognitive abilities and psychosocial functioning in BD was previously reported to be similar to that seen in schizophrenia (Depp et al., 2012). This may exacerbate the challenges that individuals with BD face in daily life, leading to greater difficulties in managing personal, social, and occupational responsibilities.

A paucity of studies have reported on the association between cognition and functioning in BD (e.g., Anaya et al., 2015; Martínez-Arán et al., 2004; Miskowiak et al., 2023; Chen et al., 2021), with most of them finding that specific cognitive abilities are predictors of functional outcomes (Bonnín et al., 2010; Lee et al., 2015; Lomastro et al., 2020; López-Villarreal et al., 2020; Ponsoni et al., 2020). While some of the studies identified executive function, verbal learning/memory (Martínez-Arán et al., 2004; Bonnín et al., 2010), verbal fluency, and working memory (Chen et al., 2021) as the predictors with the most impact on psychosocial functioning, others found that sustained attention and psychomotor speed are also important within this context (Lee et al., 2015; López-Villarreal et al., 2020). To strengthen the knowledge on these complex associations, the current study aimed to investigate the cross-sectional association between cognitive abilities and functioning. Based on previous findings, it was hypothesized that (a) there would be a significantly positive relationship between the cognitive abilities (a) attention/psychomotor speed, (b) verbal learning/memory, and (c) executive function and psychosocial functioning. Moreover, it was hypothesized that (d) attention/psychomotor speed, (e) verbal learning/memory, and (f) executive function can predict psychosocial functioning, independently of age, sex, and educational level.

## 2 Materials and methods

### 2.1 Sample

Participants were recruited and examined at the research unit and outpatient center for BD at the Medical University Graz, Clinical Department of Psychiatry and Psychotherapeutic Medicine. The current study was part of the ongoing longitudinal BIPFAT/BIPLONG study, which aims to investigate the actual and lifetime psychiatric history of BD and its association with lifestyle, metabolism, brain function, and cognition. To determine these aspects, psychiatric symptomatology, psychiatric and somatic comorbidities, anthropometric measurements, fasting blood samples, neuropsychological tests, electroencephalogram measurements, magnetic resonance imaging of the brain, and psychological questionnaires are administered semi-annually. For more information about the study design and preliminary longitudinal results see previously published reports (e.g., Dalkner et al., 2021; Fellendorf et al., 2021; Liebing et al., 2023; Queissner et al., 2024; Reininghaus et al., 2022).

In total, data from 349 individuals with a BD diagnosis were collected since the start of the study in 2012. The inclusion criteria were a diagnosis of BD, which was verified by the structured clinical interview for DSM-IV (Wittchen et al., 1997), age between 18 and 70 years, and a premorbid intelligence quotient of ≥80. Moreover, individuals were required to be euthymic at the time of testing, which was examined using the Hamilton Rating Scale for Depression (HAMD) (Hamilton, 1960) and the Young Mania Rating Scale (YMRS) (Young et al., 1978). In the current study, a HAMD score ≤ 11 and a YMRS score ≤ 8 at the time of the examination denoted a state of euthymia. Individuals were excluded if their medical history showed a diagnosis of dementia, or severe medical or neurological comorbidities such as active cancer, chronic obstructive lung disease, rheumatoid arthritis, systemic lupus erythematosus, Parkinson’s disease, Huntington’s disease, and multiple sclerosis. Overall, one individual was excluded due to a premorbid intelligence quotient of ≤80, and 95 individuals were excluded because they were not euthymic at the measurement time. Data of 43 individuals were excluded due to missing GAF scores. Thus, the final sample size comprised *N* = 210 individuals [101 female, 109 male; *M*_(age)_ = 44.47; *SD*_(age)_ = 14.25]. A sensitivity power analysis for *F*-tests (linear multiple regression analyses) using G*Power (version 3.1) (Faul et al., 2007) indicated that, when considering this sample size, an *α* of 5%, and a power of 80%, the analyses were able to detect a minimum effect size of *f*^2^ = 0.05. All participants provided written informed consent before participating in the study. The study was approved by the local ethics committee (Medical University of Graz, Austria; EC-number: 25–335 ex 11/12) and conducted in accordance with the Declaration of Helsinki.

### 2.2 Materials

#### 2.2.1 Cognitive domains

Attention/psychomotor speed. To calculate the cognitive composite score of attention and psychomotor speed, the *Trail Making Test* (TMT) (Reitan, 1958) was administered. Participants were asked to connect digits in an ascending order [part A of the TMT (TMT-A)]. The outcome measure of the TMT-A is the time to correctly complete the task. Moreover, the *Digit Span Test* [forward recall; subtest of the Wechsler Adult Intelligence Scale 4th edition (WAIS-IV) Wechsler, 2008] was administered, in which participants were asked to recall sequences of digits in the same order as previously read to them. The outcome measure of this test is the number of correctly recalled digit sequences.

Verbal learning/memory. To create the cognitive composite score of verbal learning and memory, the German version of the *California Verbal Learning Test* (CVLT) (Niemann et al., 2008) was used. Participants were instructed to memorize a list of 16 nouns drawn from four different semantic categories. This list was read to them five times in a fixed order (list A) and they were asked to recall as many words as possible in any order after each repetition (recall trials 1–5). Subsequently, a list of distractor words (list B) was presented, followed by free recall of list B. Hereafter, free and cued recalls of list A were tested immediately (short-delay free recall, short-cued recall), and again after 20 min (long-delay free recall, long-delay cued recall). Within the cued recalls, participants were prompted with the semantic word category. Finally, they were presented with a recognition task consisting of 44 words from either list A or other distractor lists. Participants were asked to indicate whether the presented word stems from list A or is a distractor word. The outcome measures of the CVLT are the numbers of correctly recalled words.

Executive function. Part B of the *TMT* (TMT-B) (Reitan, 1958) was used to measure the cognitive domain of executive function. Participants were asked to connect digits and letters in an ascending order. The outcome measure for the TMT-B was the time to correctly complete the task. Moreover, the difference between TMT-B and TMT-A was calculated to assess task-switching ability, which is part of the executive function. Further, the *Digit Span Test* (backward recall; Wechsler, 2008) was administered, in which participants were asked to recall number sequences in the reverse order, as previously read to them. The outcome measure for the Digit Span Test is the sum of correctly recalled digits. Moreover, the *Digit Symbol Substitution Test* [subtest of the Wechsler Adult Intelligence Scale 4th edition (WAIS-IV); Wechsler, 2008] was administered, which requires matching symbols to numbers according to a key located on top of the page. Participants were asked to copy the symbols into spaces below a row of numbers. The outcome measure for this test is the number of correctly copied symbols within the allowed time of 120 s.

#### 2.2.2 Clinical parameters

Global Assessment of Functioning (GAF) (American Psychiatric Association, 2002). The GAF is a summary score, which measures overall psychosocial impairment caused by mental disorders. The score was assigned by medical professionals or clinical psychologists based on the information gathered during the collection of anamnestic data and the observation of the participant’s behavior during the testing session. GAF scores range from 0 to 100, with higher scores indicating better functioning.

### 2.3 Procedure

Participants were recruited via the outpatient center of the Clinical Division of Psychiatry and Psychotherapeutic Medicine, Medical University of Graz, and were invited semi-annually for the testing sessions. However, only data of the first measurement time were used for this study. First, anthropometric and fasting biochemical measurements, which were performed for the superordinate research project, anamnestic data, and psychiatric history (e.g., BD type I/type II, illness duration, history of suicidal behavior, history of psychosis, number of affective episodes, age of onset, mood stabilizing medication, psychiatric and somatic comorbidities) were assessed by trained research staff. Subsequently, the neuropsychological test battery was administered, followed by questionnaires regarding personality and lifestyle, which were not part of the current analysis. Finally, functioning was evaluated by medical professionals or clinical psychologists using the GAF, among other psychiatric external assessments (e.g., YMRS, HAMD). Overall, the entire testing session took 2–3 h.

### 2.4 Statistical analysis

Data were analyzed using IBM SPSS Statistics (version 29).^1^ To create cognitive composite scores for attention/psychomotor speed, verbal learning/memory, and executive function, the approach outlined by Hasan et al. (2016) was applied: Initially, measures of reaction time (in which higher scores indicate inferior performance) were multiplied by −1 to ensure that higher scores are indicative of enhanced performance across all variables. Secondly, all variables were transformed to standardized *z*-scores, which were summed up for each of the three domains according to their allocation to a specific cognitive domain (see Table 1). However, most of these scores exhibited non-normal distributions, which led to a renewed *z*-transformation of the scores using Rankit’s formula, followed by a summation according to the domain allocation.

**Table 1:** Allocation of the neuropsychological tests scores to the cognitive domains.

Attention/Psychomotor speed	
	TMT-A
	Digit Span Test forward^a^
Verbal learning/memory	
Verbal learning	CVLT sum 1–5
Short-delay free recall	CVLT immediately recalled words
Long-delay free recall	CVLT recalled words after 20 min
Recognition	CVLT recognition
Executive function	
Working memory	Digit Span Test backwards^a^
	Digit Symbol Test^a^
Task switching	TMT-B
	TMT-B minus TMT-A

TMT, Trail Making Test (A/B) (Reitan, 1958); CVLT, California Verbal Learning Test (Niemann et al., 2008).

a Subtest of the WAIS-IV (= Wechsler Adult Intelligence Scale – fourth edition; Wechsler, 2008).

To test our hypotheses, three hierarchical linear regression analyses were conducted, using the GAF score as dependent variable and each of the cognitive composite scores as independent variables. Moreover, to determine possible influencing factors, Bonferroni-corrected bivariate Pearson- and Spearman correlation analyses between the cognitive composite scores, the GAF score, and the variables age, sex, and education were conducted beforehand. If any of these variables showed a significant relationship with either the cognitive composite score or the GAF score, they were considered as covariates and entered into the hierarchical regression analysis in a first step. Moreover, for the respective regression analysis, missing values in the cognitive composite scores were temporarily excluded. Assumptions to conduct all analyses were fulfilled, unless otherwise noted. Data, data codes, and a list of variables can be accessed via https://doi.org/10.17605/OSF.IO/ZDM45.

## 3 Results

### 3.1 Descriptive statistics

Table 2 provides a detailed description of the study sample and its psychometric properties.

**Table 2:** Descriptive analysis of the sample and psychometric properties.

Sex		
Male	109 (51.90%)	
Female	101 (48.10%)	
Age	*M* = 44.47	*SD* = 14.25
Education		
No formal education	6 (2.86%)	
Formal education	82 (39.05%)	
Secondary school diploma	32 (15.24%)	
High school diploma	87 (41.44%)	
No statement	3 (1.43%)	
Current job		
No employment	119 (56.67%)	
Casual employment	1 (0.48%)	
Part-time employment	25 (11.90%)	
Full-time employment	49 (23.33%)	
No statement	16 (7.62%)	
Type of bipolar disorder		
Bipolar type I	131 (62.4%)	
Bipolar type II	75 (35.7%)	
No statement	4 (1.9%)	
Current medication		
Lithium [yes]	76 (36.20%)	
Atypical antipsychotics [yes]	104 (49.50%)	
Typical antipsychotics [yes]	22 (10.50%)	
Anticonvulsants [yes]	52 (24.80%)	
Selective Serotonin Reuptake Inhibitors [yes]	47 (22.40%)	
Trycyclic antidepressants [yes]	6 (2.90%)	
Other antidepressants [yes]	64 (30.50%)	
Comorbid mental disorders		
Psychosis	38 (18.10%)	
Panic disorder	29 (13.80%)	
Obsessive-compulsive disorder	9 (4.30%)	
Post-traumatic stress disorder	8 (3.80%)	
Age at first symptoms	*M* = 24.65	*SD* = 11.12
Age at diagnosis	*M* = 36.57	*SD* = 13.11
Illness duration	*M* = 20.07	*SD* = 12.44
Number of depressive episodes	*M* = 12.38	*SD* = 14.16
Number of manic episodes	*M* = 7.78	*SD* = 9.12
GAF	*M* = 68.90	*SD* = 13.01
TMT-A raw score	*M* = 35.87	*SD* = 15.22
TMT-A standardized score (T)	*M* = 46.43	*SD* = 7.83
TMT-B score	*M* = 85.42	*SD* = 46.56
TMT-B standardized score (T)	*M* = 45.59	*SD* = 8.14
CVLT total sum 1–5	*M* = 50.62	*SD* = 13.03
CVLT total sum 1–5 standardized (T)	*M* = 43.66	*SD* = 11.43
CVLT immediate recall score	*M =* 10.28	*SD =* 3.46
CVTL immediate recall score standardized (T)	*M =* 43.31	*SD =* 10.05
CVLT delayed recall score	*M =* 11.03	*SD =* 3.54
CVLT delayed recall score standardized (T)	*M =* 44.31	*SD =* 10.62
CVLT recognition score	*M =* 15.06	*SD =* 1.34
CVLT recognition score standardized (T)	*M =* 49.01	*SD =* 7.95
Digit Symbol Test^a^ score	*M =* 62.84	*SD =* 16.62
Digit Symbol Test^a^ score standardized (T)	*M =* 44.02	*SD =* 10.64
Digit Span Test^a^ forward score	*M =* 9.09	*SD =* 1.93
Digit Span Test^a^ backward score	*M =* 6.17	*SD =* 2.34
Digit Span Test^a^ sum score standardized (T)	*M =* 60.25	*SD =* 13.89

M, Mean; SD, Standard deviation. *N* = 210. GAF, Global Assessment of Functioning; TMT, Trail Making Test (A/B) (Reitan, 1958); CVLT, California Verbal Learning Test (Niemann et al., 2008).

a Subtest of the WAIS-IV (= Wechsler Adult Intelligence Scale – fourth edition; Wechsler, 2008). T = T-score (average performance between 40 and 60).

### 3.2 Pearson- and Spearman correlation analyses

The correlation analyses indicated that the GAF score was significantly correlated with all cognitive composite scores, however, after applying the Bonferroni correction, only the correlations of attention/psychomotor speed and verbal learning/memory remained significant. Moreover, age was significantly negatively correlated, and education was significantly positively correlated with all cognitive composite scores, indicating that higher age and lower educational level are associated with lower cognitive abilities in all domains. Finally, sex was significantly correlated with verbal learning/memory, indicating that women exhibited greater cognitive abilities within this domain than men (see Table 3 for exact values).

**Table 3:** Pearson- and Spearman correlation analyses of the cognitive composite scores, Global Assessment of Functioning (GAF) score, as well as age, sex, and education.

Variable	GAF score	Attention	Memory	Executive	Age	Sex	Education^a^
GAF score	1	0.24**	0.20**	0.20	−0.14	0.07	0.16
Attention		1	0.35***	0.70***	−0.36***	0.02	0.32***
Memory			1	0.36***	−0.42***	0.21**	0.22**
Executive				1	−0.45***	−0.05	0.45***
Age					1	0.01	−0.21***
Sex						1	0.01
Education							1

*N* = 210. Attention = Cognitive composite score “Attention/psychomotor Speed.” Memory = Cognitive composite score “Verbal learning/memory.” Executive = Cognitive composite score “Executive function.” Sex: 1 = male, 2 = female. Results are adjusted using the Bonferroni-correction at an α-level of .008 (0.05/7). Significant results are printed in bold. **p < 0.01, ***p < 0.001.

a Spearman correlation analyses.

### 3.3 Hierarchical regression analyses

The hierarchical regression analysis calculating the prediction of the GAF score by attention/psychomotor speed and the control variables age and education showed that all predictors were able to collectively explain 8.7% of the variance in the GAF score. The control variables entered in a first step accounted for 6.1% of the variance, with only education contributing significant unique variance to the prediction of the GAF score. The inclusion of attention/psychomotor speed in a second step explained additional 2.6% of the variance, however, this finding did not reach statistical significance, and thus this cognitive composite score does not meaningfully contribute to functioning as measured by the GAF. Hence, only education was able to explain significant variance in functioning within this model. Table 4 shows the results of the analysis.

**Table 4:** Hierarchical regression results for attention/psychomotor speed predicting psychosocial functioning as measured by the Global Assessment of Functioning (GAF).

Variable	*B*	95% CI_(B)_		*SE_B_*	*β*	*R* ^2^	*ΔR* ^2^
		LL	UL				
Step 1						0.061	0.061*
Constant	57.11***	44.81	69.41	6.13			
Age	−0.04	−0.18	0.11	0.07	−0.04		
Education	2.18**	0.60	3.75	0.80	0.24		
Step 2						0.087	0.026
Constant	58.14***	45.91	70.37	6.18			
Age	0.01	−0.14	0.16	0.08	0.01		
Education	1.66*	0.01	3.31	0.83	0.18		
Attention/psychomotor speed	1.53	−0.05	3.11	0.80	0.18		

*N* = 135. CI, Confidence interval; LL, Lower limit; UL, Upper limit. *p < 0.05, **p < 0.01, ***p < 0.001. Significant results are printed in bold.

Moreover, the hierarchical regression analysis administered to predict the GAF score from verbal learning/memory and the control variables age, education, and sex, revealed that the predictors explained 6.3% of the total variance in the GAF score. Specifically, the control variables entered in the first step accounted for 5.1% of the variance, but only the variable age made a significantly negative unique contribution to the GAF score, indicating that higher age predicts lower psychosocial functioning. Further, the inclusion of verbal learning/memory in a second step explained additional 1.2% of the incremental variance, but this result did not reach statistical significance. This indicates that verbal learning/memory does not account for variance in functioning beyond the considered control variables (see Table 5 for exact values).

**Table 5:** Hierarchical regression results for verbal learning/memory predicting psychosocial functioning as measured by the Global Assessment of Functioning (GAF).

Variable	*B*	95% CI_(B)_		*SE_B_*	*β*	*R^2^*	*ΔR^2^*
		LL	UL				
Step 1						0.051	0.051*
Constant	65.78***	53.73	77.82	6.11			
Age	−0.14*	−0.28	−0.00	0.07	−0.15		
Education	1.07	−0.30	2.45	0.70	0.11		
Sex	2.37	−1.33	6.08	1.88	0.09		
Step 2						0.063	0.012
Constant	65.64***	53.64	77.65	6.09			
Age	−0.09	−0.24	0.05	0.08	−0.10		
Education	0.94	−0.44	2.32	0.70	0.10		
Sex	1.67	−2.14	5.48	1.93	0.06		
Verbal learning/memory	0.51	0.16	1.17	0.34	0.12		

*N* = 188. CI, Confidence interval; LL, Lower limit; UL, Upper limit. *p < 0.05, ***p < 0.001. Significant results are printed in bold.

Finally, the hierarchical regression analysis, which was conducted to predict the GAF score from executive function and the control variables age and education, revealed that the predictors collectively explained 6% of the variance in the GAF score. In particular, the control variables entered in a first step accounted for 4.7% of the total variance, however, only education made a significantly positive unique contribution to the GAF score. This indicates that higher education predicts better psychosocial functioning as measured by the GAF score. The consideration of executive function in a second step accounted for another 1.3%, however, this finding lacks statistical significance. Thus, executive function is not able to explain significant variance in psychosocial functioning beyond the considered control variables. Table 6 shows all results of this analysis.

**Table 6:** Hierarchical regression results for executive function predicting psychosocial functioning as measured by the Global Assessment of Functioning (GAF).

Variable	*B*	95% CI_(B)_		*SE_B_*	*β*	*R^2^*	*ΔR^2^*
		LL	UL				
Step 1						0.047	0.047*
Constant	58.87***	46.50	71.34	6.30			
Age	−0.04	−0.19	0.11	0.08	−0.05		
Education	1.83*	0.24	3.43	0.70	0.20		
Step 2						0.060	0.013
Constant	59.92***	47.39	72.45	6.33			
Age	0.01	−0.16	0.17	0.08	0.01		
Education	1.34	−0.41	3.08	0.88	0.15		
Executive function	0.56	−0.26	1.38	0.42	0.14		

*N* = 130. CI, Confidence interval; LL, Lower limit; UL, Upper limit. *p < 0.05, ***p < 0.001. Significant results are printed in bold.

## 4 Discussion

The current study investigated the association and prediction of psychosocial functioning by the cognitive domains attention/psychomotor speed, verbal learning/memory, and executive function. Our cross-sectional analysis showed a significantly positive association between attention/psychomotor speed, verbal learning/memory and functioning, indicating that greater abilities within these domains are related to better functioning. However, when considering the control variables age, sex, and education within hierarchical regression analyses, no cognitive domain was able to explain significant unique variance in functioning. This reflects that cognitive abilities are not predictors of psychosocial functioning beyond age, sex, and education in individuals with BD. Instead, other factors are more likely to explain psychosocial functioning of patients with psychiatric disorders, such as emotion regulation, social support, and illness-related variables. In addition, the self-assessment of cognitive deficits has also recently been linked to functioning and has been identified as an independent predictor of functional outcomes in schizophrenia (Silberstein and Harvey, 2019).

However, our hypotheses, which state that there is a significantly positive relationship between the cognitive domains (a) attention/psychomotor speed, (b) verbal learning/memory, (c) executive function and psychosocial functioning, can partly be confirmed by our results. Our finding of a positive association between attention/psychomotor speed, verbal learning/memory, and functioning is in line with previous studies, which found that impaired abilities in the domains verbal learning/memory (Bonnín et al., 2010) and attention (Jaeger et al., 2007), specifically sustained attention, were related to reduced functional disability (Lee et al., 2015). Moreover, another study found that lower performance in verbal memory, working memory, verbal fluency, and processing speed was associated with decreased functioning in BD (Sanchez-Moreno et al., 2018). Other studies reported that verbal memory represents a core deficit in BD and is one of the best predictors of functioning (Martínez-Arán et al., 2004), specifically occupational functioning (Burdick et al., 2010). The finding that none of the cognitive domains predicted functioning beyond the control variables age, sex, and education contradicts our hypotheses (d-f) and the existing literature. Most previous studies point toward a neurocognitive impairment as a main predictor of psychosocial functioning (for a review, see Keramatian et al., 2021). It should be noted that psychosocial functioning is a complex construct influenced by various non-cognitive factors, such as emotional regulation (Van Rheenen and Rossell, 2014), social support (Wang et al., 2018), illness severity (Goldstein et al., 2009; Schaub et al., 2011), and psychosocial treatment (Reinares et al., 2014). It is possible that these factors play a more dominant role in determining functional outcomes (Depp et al., 2012), thereby diminishing the predictive value of cognitive abilities in our regression analyses.

Another explanation for the non-significant regression analyses results might be the use of a single global functioning scale (the GAF as an external rating) and the creation of overarching cognitive composite scores. Studies that found an effect of cognitive abilities on functional outcomes often used measurements, which differentiated in more detailed aspects of both functioning (e.g., functioning in employment, relationship, and independent living, such as the Functioning Assessment Short Test (Rosa et al., 2007) or Work and Social Adjustment Scale (Mundt et al., 2002)) and/or cognitive domains (e.g., psychomotor speed, sustained attention; Lee et al., 2015; Flaaten et al., 2024). Possibly, the lack of differentiation into the sub-facets of both functioning and cognition might have led to a non-significant association. In addition, the participants in our sample reached test scores within 1.5 standard deviations below and above the normative performance of each test (*T*-scores of all cognitive tests ranged between *M* = 43.31–60.25 and *SD* = 7.83–13.89). This indicates that, on average, our sample showed unimpaired cognitive performance, which might have contributed to the non-significant association between cognitive abilities and psychosocial functioning. We also included individuals from our outpatient clinic, who might have a greater level of functioning, as reflected by a mean GAF score of 68.90 (*SD* = 13.01). Investigating our hypotheses in a sample with a greater range of functioning might have led to different results. Moreover, we only used data from individuals, who met the criteria for current euthymia. Other studies, which found that cognition predicts functioning in BD, analyzed data of individuals in a current affective episode (e.g., Platania et al., 2023; Torres et al., 2011; Karantonis et al., 2020), which might have led to different study outcomes. One study, which investigated the effect of clinical and cognitive variables on functioning at two measurement times in a five-year interval, reported that verbal memory at baseline and change in sustained attention during follow-up explained only 6–10%, but clinical variables (e.g., number of hospitalizations) explained up to 30% of the variance in psychosocial functioning (López-Villarreal et al., 2020). This strengthens again the assumption that other variables might exert a greater influence on functioning than cognition, such as clinical course, medication, life events, or coping styles (Fletcher et al., 2014; Sato et al., 2018; Miskowiak et al., 2023). These findings are in line with the results of the current study, which depict that demographic variables such as age and education play a more important role in explaining variance in functioning than cognitive abilities.

### 4.1 Limitations and implications

Our results should be interpreted with caution due to the following limitations. First, a major limitation is the sole use of the GAF score as representative of functioning. Using a single score of functioning might not adequately depict the distinct functioning aspects, which might otherwise be associated with cognition. Moreover, the GAF score does not address age- or sex-specific functional issues and was reported to be influenced by factors other than functional impairment (e.g., symptom severity; Bacon et al., 2002; Carrión et al., 2019). Future research should thus use additional measures of functioning, which differentiate between various aspects of functioning [e.g., Specific Level of Functioning scale (SLOF) (Schneider and Struening, 1983); UCSD (University of California, San Diego) performance-based skills assessment battery (UPSA) (Patterson et al., 2001)], and measure functioning independently of sex, age, and psychiatric symptoms (e.g., Global Functioning: Social and Role Scale; Cornblatt et al., 2007). Moreover, informant-rated or self-report measurement tools should be additionally used to assess cognitive functioning, such as the Everyday Cognition Scale (ECog) (Farias et al., 2008) or the Cognitive Functioning Self-Assessment Scale (CFSS) (Annunziata et al., 2012), since they might have an effect on the link between objective cognitive abilities and functioning. Secondly, the lack of analysis of control group data prevents a comparison between individuals with BD and mentally healthy controls. Third, the cross-sectional nature of the study prevents from drawing conclusions about causality. Finally, the results can only be interpreted for individuals with BD during euthymia, which limits the generalizability of the results.

Despite these caveats, our findings are relevant to both research and clinical contexts. The results reflect that functioning, cognition, and BD are multifaceted constructs, which need to be investigated under the consideration of several different determinants. Since functioning remains an important concept for individuals with BD, their caregivers, and treating mental health professionals, future research should continue to investigate this matter. When more data will be available, we will also re-analyze the long-term relationship between cognitive ability and level of functioning over a longer time interval in the BIPLONG cohort. Additionally, we have initiated the process of having our patients with BD assess their own cognitive abilities, as introspective accuracy has been established as an independent predictor of functioning in schizophrenia (Silberstein and Harvey, 2019). This approach may provide valuable insights into how self-perceptions of cognitive deficits correlate with actual functional outcomes in individuals with BD.

## 5 Conclusion

This study investigated the relationship between cognitive domains and psychosocial functioning in individuals with BD, revealing a positive association between attention/psychomotor speed, verbal learning/memory, and functioning. However, when controlling for age, sex, and education, these cognitive domains did not significantly predict functioning, which highlights the predominant role of demographic factors in this model. These factors are known to have a substantial impact on both cognitive performance and functional outcomes, potentially reducing the unique predictive power of cognitive domains when considered together, which needs to be considered in further studies in this area. To conclude, while our findings support prior research, which demonstrates an association between cognitive abilities and functioning, they also challenge existing literature that refers to cognition as a main predictor of functioning. Future research should incorporate more detailed functional and cognitive assessments and consider clinical variables to clarify the complex mechanisms between cognition and psychosocial functioning. Understanding these relationships is essential for developing targeted interventions that facilitate daily life in individuals with BD.

## Data Availability

The datasets presented in this study can be found in online repositories. The names of the repository/repositories and accession number(s) can be found at: Open Science Framework repository (https://doi.org/10.17605/OSF.IO/ZDM45).

## Footnotes

^1^ www.ibm.com/spss
